# Host-Induced Gene Silencing of *MoAP1* Confers Broad-Spectrum Resistance to *Magnaporthe oryzae*

**DOI:** 10.3389/fpls.2019.00433

**Published:** 2019-04-09

**Authors:** Xiao-Yi Guo, Yan Li, Jing Fan, Hong Xiong, Fu-Xian Xu, Jun Shi, Yi Shi, Ji-Qun Zhao, Yi-Fu Wang, Xiao-Long Cao, Wen-Ming Wang

**Affiliations:** ^1^Rice and Sorghum Research Institute, Sichuan Academy of Agricultural Sciences/Key Laboratory of Southwest Rice Biology and Genetic Breeding, Ministry of Agriculture, Deyang, China; ^2^Rice Research Institute, Sichuan Agricultural University, Chengdu, China; ^3^Mianyang Academy of Agricultural Sciences, Mianyang, China

**Keywords:** host-induced gene silencing, rice blast, small interfering RNA, *MoAP1*, resistance

## Abstract

Rice blast caused by *Magnaporthe oryzae* (*M. oryzae*) is a major threat to global rice production. In recent years, small interference RNAs (siRNAs) and host-induced gene silencing (HIGS) has been shown to be new strategies for the development of transgenic plants to control fungal diseases and proved a useful tool to study gene function in pathogens. We here tested whether *in vitro* feeding artificial siRNAs (asiRNAs) could compromise *M. oryza*e virulence and *in vivo* HIGS technique could improve rice blast resistance. Our data revealed that silencing of *M. oryzae MoAP1* by feeding asiRNAs targeting *MoAP1* (i.e., asiR1245, asiR1362, and asiR1115) resulted in inhibited fungal growth, abnormal spores, and decreased pathogenicity. Among the asiRNAs, asiR1115 was the most inhibitory toward the rice blast fungus. Conversely, the asiRNAs targeting three other genes (i.e., *MoSSADH*, *MoACT*, and *MoSOM1*) had no effect on fungal growth. Transgenic rice plants expressing RNA hairpins targeting *MoAP1* exhibited improved resistance to 11 tested *M. oryzae* strains. Confocal microscopy also revealed profoundly restricted appressoria and mycelia in rice blast-infected transgenic rice plants. Our results demonstrate that *in vitro* asiRNA and *in vivo* HIGS were useful protection approaches that may be valuable to enhance rice blast resistance.

## Introduction

Rice blast, caused by the fungal pathogen *Magnaporthe oryzae*, is one of the most destructive rice diseases, potentially leading to considerable economic losses for the global grain industry. It is estimated that annual rice harvests are decreased by 10–30% because of this disease (Dolgin, 2009). Therefore, it is imperative that the effective disease control strategies are developed. It is now clear that, in essence, rice has two branches of innate immune system [pathogen-associated molecular pattern- (PAMP-), triggered immunity (PTI), and effector-triggered immunity (ETI)] against *M. oryzae* (Chen and Ronald, 2011). PTI is mediated by plant pattern recognition receptors (PRRs), such as CEBiP, LYP4, LYP6, and to recognize the pathogen-derived PAMP chitin (Chandran et al., 2019). ETI is activated upon recognition of effectors from *M. oryzae* by the nucleotide binding and leucine rich repeat (NBS-LRR) type resistance (R) proteins (Chen and Ronald, 2011). So far, more than 100 rice blast *R* genes have been identified, of which more than 30 genes were isolated (Wang et al., 2017). All of the cloned *R* genes encode NBS-LRR proteins, except *Pid2* and the recessive *pi21* mutant. *Pid2* encodes a β-lectin receptor-like kinase (Chen et al., 2006). The wild type *Pi21* encodes an uncharacterized proline-rich protein containing a metal-binding domain (Fukuoka et al., 2009). Notably, several recent studies have reported that blast-resistance can be trade-off with yield penalty. For example, the interconnected two NBS-LRR receptors, PigmR and PigmS, balance high rice blast disease resistance, and yield traits via an epigenetic regulatory mechanism (Deng et al., 2017). The transcription factor ideal plant architecture 1 (IPA1) switches DNA binding specificity to regulate the expression of two down-stream genes via phosphorylation upon infection of *M. oryzae* to improve both blast disease resistance and grain yield (Wang et al., 2018). In addition, some microRNAs have been identified to be important factors in fine-tuning the trade-off between blast disease resistance and yield-traits (Chandran et al., 2019). However, *R* gene-mediated resistance is easily evaded by *M. oryzae* due to the high race-specificity of *R* genes and the high frequency of race variation in *M. oryzae* population. Thus, new approaches leading to broad-spectrum resistance are urgently needed to control the blast disease in rice production.

RNA interference (RNAi) is a conserved integral gene regulatory process in most eukaryotes (Fire, 2008). This process typically involves the cleavage of a double-stranded RNA (dsRNA) into small-interfering RNA (siRNA) homologs to a target sequence for the subsequent silencing of the genes containing that sequence. RNAi is an important pathway for functional genomics researches in many different organisms such as worms, protists, fungi, animals, plants and human (Harborth et al., 2001; Li et al., 2010; Zhu et al., 2017). The growing evidence have indicated that RNAi technology can be used in crop protection strategies (Nunes and Dean, 2012; Vinay et al., 2016). Especially, the RNAi has been used commercially to engineer virus-resistant plants via the expression of viral sequences as transgenes (Frizzi and Huang, 2010), indicating that the host-induced gene silencing (HIGS) is an RNAi-based technology that emerged as a powerful tool for generating transgenic plants to control fungal diseases and study the function of candidate pathogenicity genes in pathogenic fungi (Nunes and Dean, 2012). This technology has been proven to be effective in the following fungal pathogens by *in planta* expression of RNAi constructs specifically silencing certain fungal genes, including *Fusarium graminearum* (Koch et al., 2013; Cheng et al., 2015), *Fusarium culmorum* (Chen et al., 2016), *Blumeria graminis* (Nowara et al., 2010; Pliego et al., 2013), *Puccinia striiformis* f. sp. *tritici* (Yin et al., 2011; Zhang et al., 2012), *Puccinia triticina* (Panwar et al., 2013), *Fusarium oxysporum* f. sp. *cubense* (Ghag et al., 2014), *Botrytis cinerea* (Wang et al., 2016), and *Bremia lactucae* (Govindarajulu et al., 2015). In recent researches, HIGS has been used to study the rice-*M. oryzae* interaction. Wang et al. (2015) cloned *M. oryzae NoxI* and *NAC* gene fragments and constructed HIGS transgenic rice plants, but they did not show whether the transgenic rice lines exhibited resistance to blast disease. Zhu et al. (2017) silenced 3 rice blast pathogenicity genes by transient BMV-HIGS system, and indicated that combining introduction of fungal gene sequences in sense and antisense orientation could significantly enhanced the efficiency of BMV-HIGS. However, they didn’t get the stable resistent transgenic rice lines.

*Magnaporthe oryzae* infects in host to obtain nutrients for its subsequent proliferation. The infection process involves multiple steps, including conidial germination, appressorium formation, penetration peg formation, and invasive hyphal growth (Wilson and Talbot, 2009). Each step is regulated by different genes, some of which have been functionally characterized. For example, *PLS1* is involved in the regulation of appressorial function that is essential for the penetration of host cells by the fungus (Clergeot et al., 2001). *PMK1* regulates appressorium formation and hyphal growth (Park et al., 2002), and *GAS1*, *GAS2* and *MST12* may function downstream of *PMK1* to regulate the expression of genes involved in appressorial penetration and hyphal growth (Park et al., 2002; Xue et al., 2002). Furthermore, *PDE1* (Balhadere and Talbot, 2001) and *MPS1* (Xu et al., 1998) influence appressorium-mediated plant infection, whereas, *RVS167* and *Las17* affect appressorial penetration (Dagdas et al., 2012). Penn et al. (2015) indicated that protein kinase C is essential for growth and development of the rice blast fungus and may play key roles in spore germination, cell wall biogenesis, polarized growth, and hyphal development. The infection process of *M. oryzae* also involves transcription factors. For example, *MoAP1* encodes an *M. oryzae* bZIP transcription factor essential for conidial production, appressorial development, aerial hyphal growth, and pathogenicity (Guo et al., 2011). *MoAP1* regulates the expression of *MoAAT*, *MoSSADH* and *MoACT*, which are important for the growth, development, and pathogenicity of *M. oryzae* (Guo et al., 2011). Additionally, *MoSOM1* and *MoCDTF1* are also transcription regulators associated with cellular differentiation of invasive hyphae during infections of the rice blast fungus (Yan et al., 2011). Therefore, disrupting the infection process via HIGS of these genes may result in the weakening or loss of pathogenicity, leading to broad-spectrum rice blast resistance.

In this study, we demonstrated that silencing of the *M. oryzae* transcription factor *MoAP1* by RNAi *in vitro* led to compromised *M. oryzae* virulence and by HIGS in transgenic rice lines led to improved blast disease resistance. Our findings may be relevant for generating new rice lines exhibiting a HIGS-based broad-spectrum resistance to rice blast disease.

## Materials and Methods

### Plant Material and Rice Blast Fungus

The *indica* rice accession Kasalath was used for transgenic analyses. The *M. oryzae* strain Guy11 was used as a reference strain. The GFP-tagged strain GZ8 was used in observation of the infection process. Eleven strains (Supplementary Table S1) stored in the Plant Pathology Laboratory (the Rice Research Institute, Sichuan Agricultural University, China) were used for disease assay.

### asiRNA Design and Synthesis

The genomic sequences for *MoAP1* (*MGG_12814*), *MoSSADH* (*MGG_01230*), *MoACT* (*MGG_15157*), and *MoSOM1* (*MGG_04708*) were download from the *M. oryzae* database^1^. Nineteen-nucleotide sequences in three different sites specific to each genes were selected as the asiRNAs candidates. The specificity of the sequences was confirmed by BLASTn against the *M. oryzae* genomic sequences to avoid off-targeting. The complementary sequences of these candidates were then designed as double strand artificial small interference RNAs (asiRNAs) (Supplementary Table S2). Double T nucleotides were added to the 3′-terminus to stabilize the asiRNAs. Then, the TT-overhang double strand twenty-one-nucleotide sequences were synthesized by Shanghai Genepharma. Co., LTD. (China).

### *In vitro* Culture of *M. oryzae* With asiRNA

Artificial siRNAs were added into solid CM medium (Choi et al., 2015) with different concentrations (i.e., 0, 50, 100, or 200 nM). Two microliter (μl) of *M. oryzae* spore suspension (1 × 10^5^ spores /ml) was inoculated on the medium and incubated at 28°C with 12-h light/ 12-h darkness. The development of spores and hypha was monitored with a microscope and the aerial hyphae growth rate (AHGR) was calculated as percentage of the amount of healthy aerial hyphae divided by the sum of healthy aerial hyphae and abnormal aerial hyphae at 1 dpi in three biological replicates. Each replicate contained at least 30 spores inoculated. After sporulation, the conidia deformity rate was calculated as percentage of the amount of healthy conidia divided by the sum of healthy conidia and abnormal conidia at 7 dpi in three biological replicates. Each replicate contained at least 30 conidia inoculated.

### Pathogenicity Assay for the Rice Blast Fungus Treated With asiRNA

The *M. oryzae* strains Guy11 were cultured on CM solid medium containing asiR1115 for 14 days at 28°C with 12-h light/ 12-h darkness. Then the spores were collected and 5 μL of spore suspension (1 × 10^5^ spores/ml) were punch-inoculated on the third leaf of TaiPei309 (TP309) from three-leaf stage seedlings. The disease phenotypes on the leaves were observed at 7 dpi. Images were taken and the lesion area was calculated as the product of the length and width of the lesions.

### qRT-PCR Assay

Three-leaves-stage transgenic rice plants were inoculated with asiR1115-treated (50 and 100 nM) *M. oryzae* spore suspension (1 × 10^5^ spores/ml). After 7 dpi, disease lesions were recorded before sampling fore RNA extraction. Total RNA was extracted from the lesions using Trizol reagent (Invitrogen). The first-strand cDNA was synthesized using the Super-Script First-strand Synthesis System (Invitrogen). A qRT-PCR assay was conducted using the SYBR Green Mix (TaKaRa) to measure relative transcript abundance. *MoPot 2* expression was normalized against that of *OsActin1* to be the internal control (Chandran et al., 2019). The qRT-PCR primers are listed in Supplementary Table S3.

### *In vitro* asiRNA-Spraying Assay

Four- to six-leaf stage TP309 seedlings were sprayed with 50 or 100 nM asiR1115 in 0.1% tween 20. Twelve hours after spraying, *M. oryzae* strain Guy11 was inoculated by punch-inoculation. The disease phenotypes on the inoculated leaves were observed at 7 dpi. Images were taken and the lesion area was measured as the product of the length and width of the lesion points.

### Construction of RNAi Vectors and Genetic Transformation

Three *MoAP1* exon fragments of 242, 255, and 247 bp containing asiR1115, asiR1245, and asiR1362 sequences, respectively, were amplified from *M. oryzae* mycelia DNA with *MoAP1*-specific primers (Supplementary Table S4). The PCR fragments were cloned into the pCR8-T plasmid [(Cat No.:K2500-20, Invitrogen), and then inserted into the RNAi vector pANDA using the recombination Gateway system (Cat No.:11791020, Invitrogen; Miki and Shimamoto, 2004) leading to constructs pANDA-*MoAP1-1115* (1115i), pANDA-*MoAP1-1245* (1245i), and pANDA-*MoAP1-1362* (1362i)]. The constructs were introduced into rice accession Kasalath via *Agrobacterium*-mediated transformation following a previous report ( Li et al., 2017). Briefly, the pANDA-*MoAP1*-RNAi plasmids (i.e., 1115i, 1245i, and 1362i) were transformed into *Agrobacterium* strain *EHA105*, and then used to infect rice callus from the mature embryo of Kasalath. Positive transformants were screened by means of hygromycin resistance analysis.

### Agrobacterium-Mediated Transient Expression Assay in *Nicotiana benthamiana*

YFP detection was assayed as previously reported (Li et al., 2017). We fused YFP with *MoAP1* at its C-terminus (*35S:MoAP1-YFP*) and inserted into *Kpn*I-*Spe*I sites of binary vector 35S-pCAMBIA1300. Then the construct was transformed into *Agrobacterium* strain GV3101 for agro-infiltration-mediated transient expression assay in *N. benthamiana*. In brief, *Agrobacterium* strain GV3101 harboring *MoAP1-YFP* constructs and *Agrobacterium* strain EHA105 harboring the indicated constructs (empty vector pANDA, 1115i, 1245i, and 1362i) were incubated at 28°C overnight in LB media containing kanamycin (50 mg/ mL) and carbenicillin (50 mg/ mL) on a table shaking at 250 rpm. The bacteria were collected by centrifuge at 800 × *g* for 5 min and resuspended in an MMA buffer (10 mM 4-Morpholineethanesulfonic acid hydrate, 10 mM MgCl_2_, 100 mM Acetosyringone). The indicated Agrobacteria were infiltrated into leaves of *N. benthamiana*. Leaves were examined at 48 h post infiltration (hpi) for image acquisition using a NikonA1 Confocal Laser Scanning Microscope (Nikon Instruments, Inc., China).

### Infection Process Assay

For observing the infection process of **M. oryzae**, we inoculated the GFP-tagged strain GZ8 on 4-cm-long leaf sheaths as described (Kankanala et al., 2007). The inoculated epidermal layer was excised and analyzed by fluorescence microscopy (Zeiss Axio Imager A2) at 36 hpi.

## Results

### The Artificial siRNAs Targeting *MoAP1* Effectively Suppressed the Growth of Aerial Hyphae *in vitro*

**FIGURE 1:**
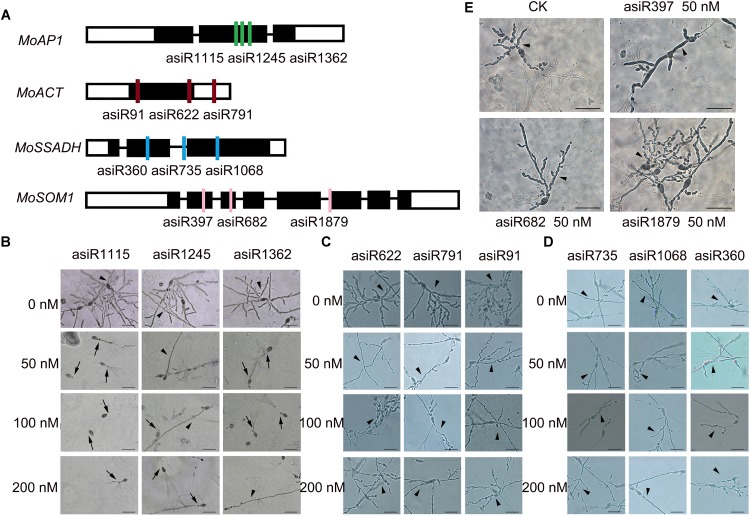
Growth of aerial hyphae of rice blast fungus on media containing artificial siRNAs (asiRNAs). **(A)** Diagrams show the target sites of the indicated artificial small interference RNAs. Black boxes denote exons. **(B)** The indicated asiRNAs targeting *MoAP1* inhibited fungal aerial hyphae growth. The culture medium was supplemented with the indicated asiRNAs targeting *MoAP1* at the indicated concentration. Control (CK) samples with 0 nM asiRNAs. Note that abnormal hyphae consisted of degraded hyphal cytoplasm and lacked melanin (arrows). Normal hyphae exhibited robust growth and had relatively high cytoplasmic melanin levels in the CK media (arrowheads). **(C–E)** The indicated asiRNAs targeting *MoACT* **(C)**, *MoSSADH* **(D)**, and *MoSOM1* **(E)** did not affect *Magnaporthe oryzae* aerial hyphae growth. Images were taken at 1 day post conidial culture. Bars = 50 μm. Similar results were obtained in at least three independent experiments.

**FIGURE 2:**
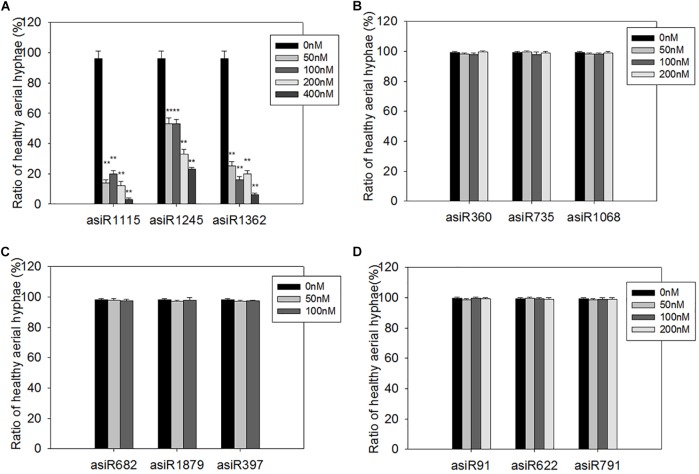
Quantification of healthy aerial hyphae on media containing different asiRNAs. **(A–D)** Quantification of healthy aerial hyphae ratio on the media containing the indicated asiRNAs targeting *MoAP1* **(A)**, *MoSSADH* **(B)**, *MoSOM1* **(C)**, and *MoACT* **(D)**. Note that the ratio of healthy aerial hyphae was substantially declined in the media containing asiRNAs targeting *MoAP1* **(A)**. While the indicated asiRNAs targeting *MoSSADH* **(B)**, *MoSOM1* **(C)**, and *MoACT* **(D)** had no inhibitory effect on fungal aerial hyphae growth. Values were means ± SD from three replicates, in each replicate, more than 30 spores of hyphae were evaluated at 1 day post conidial culture. Asterisks (^∗∗^) above the bars indicate significant differences at *P* < 0.01 between the indicated treatment and control as determined by student’s *t-test*. All the experiments were repeated at least three times with similar results.

To screen a small RNA that could be used in HIGS transgenic analysis, we first tested whether the *in vitro* treatment of asiRNAs affects *M. oryzae* growth in an axenic culture. To this end, we designed and synthesized asiRNAs targeting *M. oryzae* transcription factor genes *MoAP1*, *MoSSADH*, *MoACT*, and *MoSOM1*, respectively (Supplementary Table S2). The sites targeted by asiRNAs in each of the 4 genes are shown in Figure 1A and the selected sequences for asiRNAs were BLASTn against the with *M. oryzae* genome sequence to make sure the asiRNAs were specific. Microscopic analyses revealed that increasing concentrations of the asiRNAs targeting *MoAP1* inhibited the growth of *M. oryzae* aerial hyphae and resulted in abnormal hyphae consisting of degraded cytoplasm and lackng melanin. Abnormal aerial hyphae were not detected in the control *M. oryzae* sample (Figure 1B). By contrast, the samples treated with asiRNAs targeting the *MoSSADH*, *MoACT*, and *MoSOM1* did not show obvious abnormal growth or development (Figure 1C–E). The AHGR was analyzed to quantify the inhibitory effects of asiRNAs. The AHGR of *MoAP1*-silenced *M. oryzae* was significantly decreased upon 50 nM asiRNA treatment, and further decreased when asiRNA concentrations were increased. Among the three asiRNAs targeting *MoAP1*, asiR1115 displayed the most inhibition on fungal aerial hyphae growth, followed by asiR1362 and asiR1245 (Figure 1B, 2A). In contrast, the asiRNAs targeting *MoACT*, *MoSSADH*, and *MoSOM1* did not affect *M. oryzae* aerial hyphae growth (Figure 1C–E, 2B–D).

These results indicated that the asiRNAs targeting *MoAP1* effectively suppressed the growth of aerial hyphae, which is consistent with the function of *MoAP1* reported previously (Guo et al., 2011).

### asiRNA Treatment Results in Conidial Deformity

**FIGURE 3:**
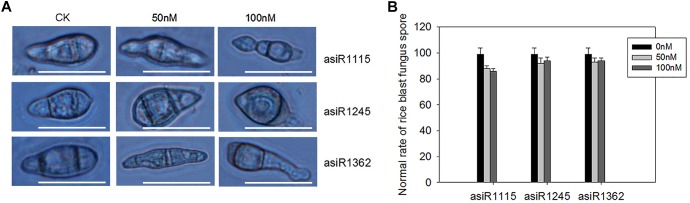
Abnormal conidial morphology formed on media containing asiRNA targeting *MoAP1*. **(A)** The abnormal conidial morphology with the indicated asiRNA targeting *MoAP1* at the indicated concentration. Bars = 25 μm. **(B)** Quantitative analysis on ratio of normal fungal conidia treated with asiRNAs. Values were means ± SD from three replicates. In each replicate, more than 30 conidia were evaluated at 7 days post conidial culture. All the experiments were repeated at least three times with similar results.

To further test whether development of *M. oryzae* conidia was affected by asiRNAs, we checked the fungal conidia treated with the asiRNAs at 7 days post conidial culture. Microscopic analyses revealed that the *in vitro* treatment with asiRNA targeting *MoAP1* resulted in malformed conidia, including elongated, and spindly morphology (Figure 3A,B). These results indicates that the three asiRNAs targeting *MoAP1* influence the conidial development.

### The asiRNA Targeting *MoAP1* Affects Fungal Colony Morphology

**FIGURE 4:**
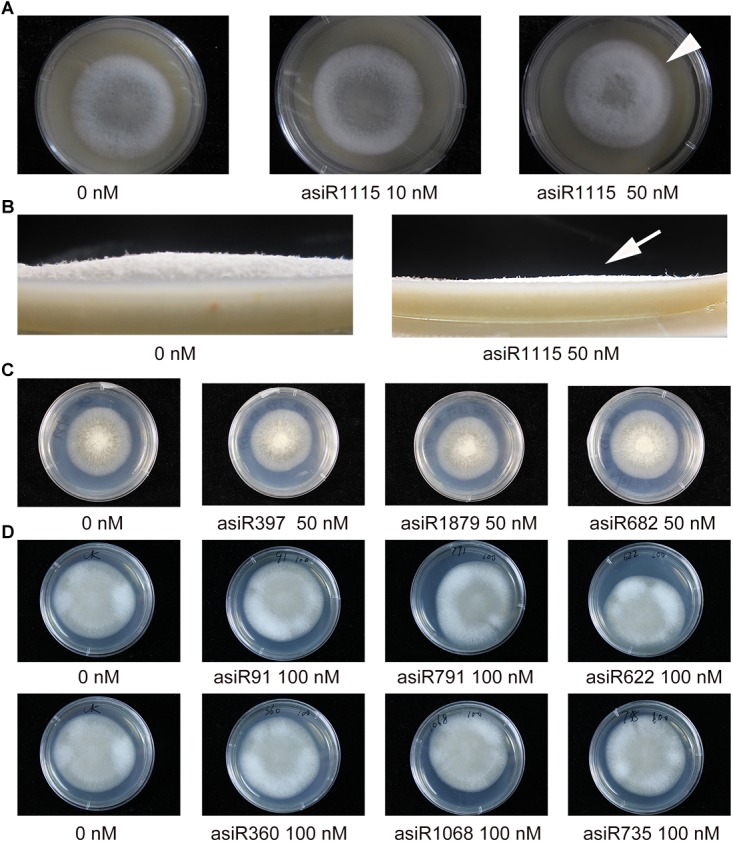
The effect of asiRNAs targeting *MoAP1* on fungal colony morphology. **(A)** The colony morphology of *M. oryzae* growing on media containing the indicated asiRNA targeting *MoAP1*. Note that the asiRNA treatments resulted in short and sparse aerial hyphae, with an obviously delayed development of outer circle of the fungal colony (arrowhead), which differed from that of the control. **(B)** Side view of the colony morphology of *M. oryzae* growing on media containing the indicated asiRNA targeting *MoAP1.* It showed decreased growth of aerial hyphae on media containing 50 nM of asiR1115 targeting *MoAP1* in comparison with that of 0 nM (arrow). **(C,D)** Colony morphology in media supplemented with different concentrations of asiRNAs targeting *MoSOM1*, *MoACT* and *MoSSADH*, respectively. Note that these asiRNAs did not affect the colony morphology. Images were taken at 7 days post mycelia colony culture. Similar results were obtained in three independent experiments.

Magnaporthe *oryzae* colony morphology was affected by different concentrations of asiR1115. Treatments with the asiRNA targeting *MoAP1* resulted in significantly decreased growth of aerial hyphae (arrows), and an obviously delayed development of outer circle of the fungal colony, which differed from that of the controls (Figure 4B). This result indicated that the asiRNAs targeting *MoAP1* effectively suppressed the growth of aerial hyphae leading to change in colony morphology. However, the asiRNAs targeting *MoACT*, *MoSSADH*, and *MoSOM1* did not affect fungal colony morphology (Figure 4C,D).

### *In vitro* Silencing of *MoAP1* by asiRNAs Resulted in Lower Virulence of *M. oryzae*

**FIGURE 5:**
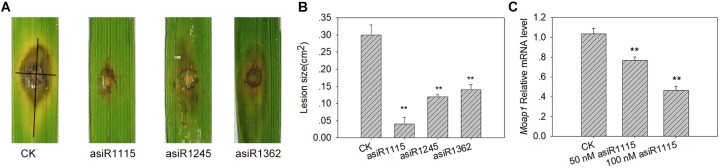
Silence of *MoAP1* by asiRNAs resulted lower virulence of *M. oryzae*. **(A)** Disease phenotypes of TP309 at 7 days post-inoculation (dpi) by asiRNA-treated *M. oryzae* strains Guy11. Rice cultivar TP309 was punch-inoculated with Guy11 (1 × 10^5^ spores/ml) cultured on media containing the indicated asiRNAs targeting *MoAP1*. **(B)** Quantitative analysis on lesion area at 7 dpi. The length and width of the symptom were measured, and the product of the length and width were designed as the area of the symptom. Black lines in lesion **(A)** were used to represent length and width. Values were means ± standard deviations (SD) of 15 disease spots. Asterisks (^∗∗^) above the bars indicate significant differences at *P* < 0.01 as determined by student’s *t-test* analysis. **(C)** The relative mRNA level of *MoAP1* in the mycelium from the lesions of **(A)** generated by asiR1115-treated Guy11 and control. The expression of *MoAP1* was determined by qRT-PCR. The relative *MoAP1* mRNA levels were measured by using the mRNA level of *M. oryzae MoAP1*. Values are means ± standard deviations (SD) of three replicates. Asterisks (^∗∗^) above the bars indicate significant differences at *P* < 0.01 as determined by student’s *t-test*. All experiments repeated three times with similar results.

Because asiRNA targeting *MoAP1* influenced the growth and development of *M. oryzae*, we asked the question whether the affected fungal conidia were virulent to rice. To this end, we inoculated susceptible rice cultivar TP309 plants with spores of the *M. oryzae* strain Guy11 cultured on the media containing asiRNAs targeting *MoAP1*. Intriguingly, all three asiRNAs-treated *M. oryzae* Guy11 resulted in compromised symptom with smaller lesion area (Figure 5A,B). Furthermore, asiR1115-treated Guy11 developed the slightest symptom and smallest lesion area (Figure 5A,B). These data indicated that all three asiRNAs targeting *MoAP1* effectively compromised the virulence of the rice blast fungus. These results were consistent with that loss-of function in *MoAP1* led to loss virulence of the fungus (Guo et al., 2011).

Next, we examined the expression of *MoAP1* in the mycelium developed on the leaf lesions which were inoculated with Guy11 cultured on the media containing asiR1115 targeting *MoAP1*. The qRT-PCR results indicated that *MoAP1* expression was significantly lower in the lesions generated by asiR1115-treated Guy11 than in the control lesions (Figure 5C), indicating that asiR1115 effectively suppressed *MoAP1* expression *in vitro* and such suppression could maintain in the subsequent fungal mass grown in rice leaves.

### AsiRNA-Treated Leaves Shows Increased Resistance to Rice Blast Fungus

**FIGURE 6:**
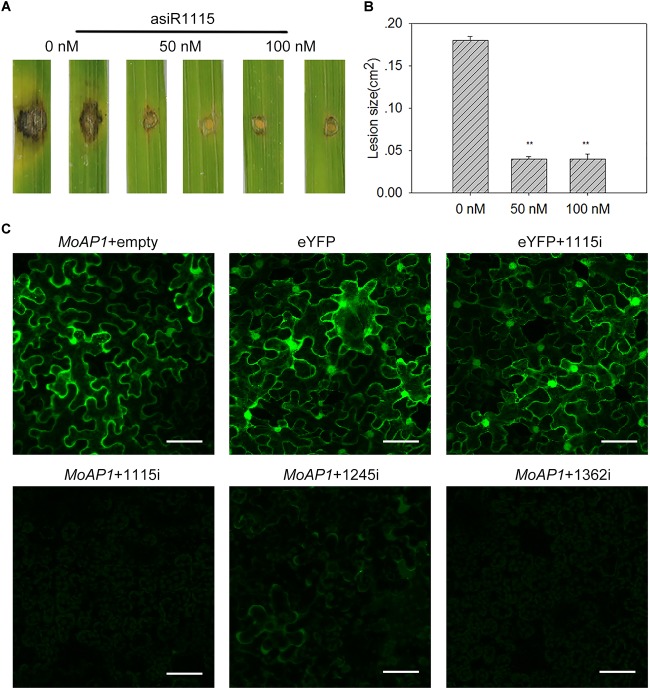
Artificial siRNAs targeting *MoAP1* inhibits the expression of *MoAP1 in vivo* and compromises *M. oryzae* virulence. **(A)** Disease phenotypes of TP309 leaves pre-treated with the indicated concentration of asiRNA1115. TP309 third leaves of seedlings were smeared by a brush soaked in 50 or 100 nM asiR1115 solution before inoculation with 5 μL of spore suspension (5 × 10^5^ spores/ml) of *M. oryzae* strain Guy11. Disease phenotype was recorded at 7 dpi **(B)** Quantitative analysis of lesion area at 7 dpi. The length and width of the symptom were measured, and the product of the length, and width were designed as the area of the symptom. Values are means ± standard deviations (SD) of 15 disease spots. Asterisks (^∗∗^) above the bars indicate significant differences at *P* < 0.01 as determined by student’s *t-test*. This experiment was repeated three times with similar results. **(C)** Confocal images show the inhibition of asiRNAs on the expression of *MoAP1*-eYFP in *N*. *benthamiana*. Transient expression of *35:MoAP1*-*eYFP* (*MoAP1*) plasmid with an empty vector (no asiRNA) in tobacco as positive control (Supplementary Figure S2). *35:MoAP1*-*eYFP* co-expressed with the asiRNAs (Supplementary Figure S1) as the test group. The eYFP and eYFP co-expressed with asiRNA1115 as the negative control. The images were taken at 2 days post infiltration. Bars = 50 μm. All experiments were repeated three times with similar results.

To test whether asiRNAs can be used on rice to improve rice resistance against *M. oryzae*, we pre-treated rice leaves with 50 or 100 nM asiR1115. Then, we inoculated the pre-treated leaves with Guy11 by punch-inoculation (Li et al., 2017). The control plants were pre-treated with water. After 7 dpi, the lesion area were measured. Our results showed that the asiR1115-pretreated leaves formed lesions much smaller than the control leaves (Figure 6A,B), indicating that asiRNA may be used as an *in vitro* treatment to enhance rice blast resistance.

### *In vivo* Suppression of *MoAP1* Gene Expression by asiRNAs in a Heterologous System

The *in vitro* data indicated that asiRNA-treatment could suppress *MoAP1* expression to decrease *M. oryzae* virulence. We then tested whether asiRNAs could silence *MoAP1 in planta*. To this end, we made constructs expressing eYFP fused with the *MoAP1* at its 5′-terminus (*35S:MoAP1-eYFP*) (Supplementary Figure S1B) and constructs expressing asiRNAs containing asiRNA1115, asiRNA1245, and asiRNA1362, respectively (Supplementary Figure S1A). Then, *35S:MoAP1-eYFP* was transiently expressed in *N*. *benthamiana* alone or together with asiRNA1115, asiRNA1245, or asiRNA1362. The YFP intensity expressed from *35S:MoAP1-eYFP* was obviously lower when co-expressed with either one of the asiRNAs than *35S:MoAP1-eYFP* alone (Figure 6C). In contrast, the YFP intensity expressed from *35S:eYFP* was unchanged when co-expressed with asiRNA1115 (Figure 6C), indicating the tested three asiRNAs could suppress *MoAP1* expression *in vivo.*

### HIGS of *MoAP1* in Transgenic Rice Results in Compromised Susceptibility

**FIGURE 7:**
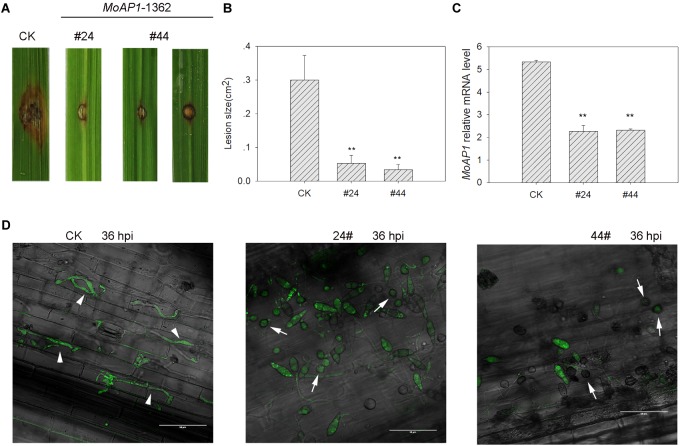
Analyses of the resistance of *MoAP1*-RNAi transgenic plants to the rice blast fungus and the *MoAP1* expression in inoculated T_1_ transgenic plants. **(A)** Blast disease assay on the indicated T1 transgenic lines. The control plants (CK) and *MoAP1*-1362 T_1_ transgenic lines were punch-inoculated with 5 μL of spore suspension (5 × 10^5^ spores mL^−1^) of *Magnaporthe oryzae* Guy11. Images were taken at 7 dpi. CK: the rice cultivar Kasalath expressing empty vector; #24 and #44: *MoAP1*-1362 T_1_ generation transgenic lines. **(B)** Quantitative analysis of lesion area at 7 dpi. The length and width of the lesions were measured, and the product of the length and width was designed as the lesion size. Values are means ± standard deviations (SD) of 15 disease spots. Asterisks (^∗∗^) above the bars indicate significant differences at *P* < 0.01 between the indicated lines and control as determined by student’s *t-test*. This experiment was repeated three times with similar results. **(C)** qRT-PCR showing that *MoAP1* expression in *MoAP1*-1362 T_1_ generation transgenic lines in comparison with control at 7 dpi. Asterisks (^∗∗^) above the bars indicate significant differences at *P* < 0.01 between the indicated lines and control as determined by student’s *t-test*. **(D)** Confocal images show the infection status of *M. oryzae* GZ8 in the indicated lines at 36 hpi. Note that appressoria (arrows) were formed and delayed to 36 hpi on *MoAP1*-1362 transgenic plants, by contrast, the invasive hyphae (arrowheads) were formed and extended to the neighboring cell on empty vector plants at 36 hpi. Bars = 20 μm. All experiments repeated three times with similar results.

Next, we tried to make rice transgenic lines overexpressing the three asiRNAs and successfully got transgenic lines containing asiR1362 (*MoAP1*-1362, Supplementary Figure S2). Then, these transgenic lines were subjected to disease assay. Positive T_1_ transgenic plants were punch-inoculated with *M. oryzae* strain Guy11. Disease phenotype was recorded at 7 dpi. As our expectation, the transgenic lines displayed infection area obviously smaller than the control plants (Figure 7A,B). Then, we examined the expression of *MoAP1* in the fungal mass collected at the infection area. Consistently with the smaller infection area, *MoAP1* expression was significantly decreased in the fungal mass collected from transgenic lines (Figure 7C), indicating HIGS of *MoAP1*. These results indicated that HIGS of *MoAP1* could improve rice resistance against *M.oryzae*.

### Cellular Responses of Transgenic Rice Lines to *M. oryzae*

To understand how HIGS of *MoAP1* leading to enhanced blast resistance, we examined the infection process of the GFP-tagged strain GZ8 by laser scanning confocal microscopy. To this end, GZ8 was inoculated on sheath of *MoAP1*-1362 transgenic plants and control plants. Then, the infection stage was recorded under fluorescence microscopy. At 36 hpi, the invasive hyphae were formed and extended to the neighboring cells on control plants. By contrast, the conidia on the transgenic lines mostly formed long germ tubes outside the host cells and stayed at appressoria-forming stage (Figure 7D), indicating failure in penetration.

### HIGS of *MoAP1* Coffers Broad-Spectrum Resistance to *M. oryzae*

**FIGURE 8:**
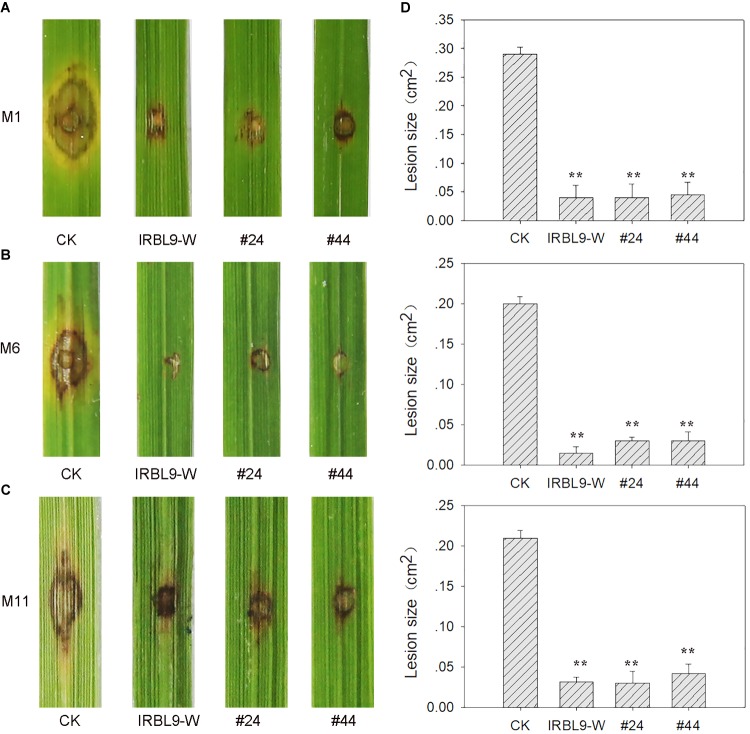
Disease resistance spectrum of transgenic plants. **(A–C)** Disease phenotype of the indicated lines punch-inoculated with *M*. *oryzae* strains M1 (Tepep) **(A)**, M6 (Zhong1) **(B)**, and M11 (089) **(C)**. Two T_3_ lines (i.e., *MoAP1*-1362 transgenic lines #44 and #24) were punch-inoculated with 5 μL of spore suspension (5 × 10^5^ spores mL^−1^) of the indicated *M*. *oryzae* strains. CK, the rice cultivar Kasalath expressing empty vector. IRBL9-W, a monogenetic rice line containing the blast resistance gene *Pi9*. Images were taken at 7 dpi. **(D)** Quantitative analysis of lesion area at 7 dpi. The length and width of the lesions were measured, and the product of the length and width was designed as the lesion size. Values are means ± standard deviations (SD) of 15 disease spots. Asterisks (^∗∗^) above the bars indicate significant differences between the indicated lines and control at *P* < 0.01 as determined by student’s *t-test*. All experiments repeated three times with similar results.

Next, we asked whether the *MoAP1*-1362 transgenic plants also were resistant to other *M. oryzae* isolates. To address this question, we inoculated *MoAP1*-1362 transgenic lines with 11 *M. oryzae* strains derived from rice fields in Ya’an, Sichuan Province, China (Supplementary Table S1). We found that the transgenic lines displayed increased resistance or compromised susceptibility to all 11 tested *M. oryzae* strains (Figure 8 and Supplementary Table S1), indicating that the *in vivo* expression of siRNAs triggered HIGS of *MoAP1* leading to increased rice resistance against rice blast disease.

## Discussion

Recent studies have shown that HIGS technology was a great strategy for protecting rice plants against the pathogenic fungi *M. oryzae*, but they didn’t get the stable resistent transgenic rice lines (Zhu et al., 2017). In this study, we demonstrated that HIGS of *M. oryzae* transcription factor gene *MoAP1* is efficient for controlling the infection of rice blast fungus. Transcription factor genes, such as *MoAP1*, *MoSSADH*, *MoACT*, and *MoSOM1* in the rice blast fungus, are important regulators of mycelial development, sporulation, and pathogenicity. we designed three asiRNAs for each gene and tested their role on fungal growth by adding them in the medium. The three asiRNAs (i.e., asiR1245, asiR1362, and asiR1115) targeting *MoAP1* all resulted in abnormal aerial hyphae, deformed spores, and decreased virulence with different inhibitory effects (Figure 1–5). AsiR1115 had the greatest inhibitory effect *in vitro*, with a minimal effective concentration of 50 nM (Figure 6). Therefore, artificial siRNA *in vitro* assay could serve as a quick prescreening to identify efficient HIGS target for stable transformation. Interestingly, the persistence of the silencing effect was found in inoculum grown on medium with *MoAP1* asiRNA. The three asiRNAs-treated *M. oryzae* Guy11 resulted in compromised symptom with smaller lesion area on rice. The possible explanation is that, the fungus cultured on medium may absorb asiRNA from the medium, which resulted in the suppressed hyphae growth and malformed conidia, including elongated, and spindly morphology, indicating that the silencing effect is hereditary and persistence. Next, we focused on asiRNAs targeting *MoAP1* to verify whether HIGS could provide resistance against the rice blast fungus *in vivo*. We constructed transgenic lines expressing siRNAs that targeted *MoAP1* based on HIGS technology. These transgenic lines exhibited enhanced resistance or compromised susceptibility when different *M. oryzae* strains were inoculated (Figure 7, 8). During infection, the fungus may be able to take up siRNAs from the plant cells, resulting in decreased *MoAP1* gene expression *via* an RNAi mechanism. As a result, the virulence of the fungus may be compromised due to the lower expression of *MoAP1*.

The HIGS system represents a potentially powerful reverse genetics tool for analyses of fungal gene functions. The effect of HIGS may differ on different genes and on different target sites of one gene. Here we found that asiRNAs targeting *MoAP1* were effective *in vitro* and HIGS of *MoAP1* worked *in vivo* (Figure 5–8). Nevertheless, the three asiRNAs targeted at different sites of *MoAP1* and the site on the most up-stream of *MoAP1* seems the best in silencing of *MoAP1* (Figure 2). However, the asiRNAs targeting the other three genes (i.e., *MoSSADH*, *MoACT*, and *MoSOM1*) did not result in any *in vitro* phenotypes (Figure 1, 2). Further investigation is needed to make out whether the asiRNA sites we selected were not work or silencing these genes did not lead to any obvious phenotypes. In fact, some genes are not suitable for HIGS because knocking-down there expression may not have effect. For example, Yin et al. (2011) chose twelve pathogenic-related genes of *P. striiformis f.* sp. *tritici or P. graminis f.* sp. *tritici* to determine whether silencing signals can be delivered to the pathogen and suppress expression of the fungal genes by HIGS. The results indicated that some candidate genes are not suitable as targets for HIGS. Govindarajulu et al. (2015) verified this conclusion. HIGS efficiency of different target genes may be related to the function of target genes and their expression patterns. Selection of certain pathogenic genes transcribed highly in the infectious hyphae may be beneficial to control rice blast disease effectively (Zhu et al., 2017). In addition, the size, sequence specificity, and position of the dsRNA are also important in considerations for HIGS (Thomas et al., 2001; Holen et al., 2002; Burch-Smith et al., 2004). The higher the homology between siRNA and the target mRNA, the better the effect of gene silencing. several siRNAs synthesized against different sites on the same target mRNA (human Tissue Factor) demonstrated striking differences in silencing efficiency. And previous studies have indicated that, if the gene fragment selection was not suitable, it may cause off-target silencing, so that no effective silent phenotype can be obtained (Xu et al., 2006; Senthil-Kumar and Mysore, 2011), and several siRNAs synthesized against different sites on the same target mRNA demonstrated striking differences in silencing efficiency (Holen et al., 2002). To achieve a better silencing effect, the length of the gene fragment inserted into the carrier should be limited to 200–350 bp (Burch-Smith et al., 2006), and using a hairpin RNA expression pattern will significant increase efficiency of gene silencing (Pflieger et al., 2008; Zhu et al., 2017). And some strategies are feasible to exploit HIGS for durable resistance. (i) using a silencing construct that targets entire gene families, (ii) multiple lines deployed in rotation, (iii) a HIGS line conferring resistance to single or multiple fungal pathogens or (iv) a combination of both classical R genes and HIGS (Nunes and Dean, 2012).

We observed phenotypes on silencing of *MoAP1*. The hyphae can grow and appressoria can form on the surface of transgenic plant leaves (Figure 7D), but are strongly inhibited *in vitro* (Figure 1B). We think the possible reason is that *in vitro* experiments, the fungus cultured on medium may absorb asiRNA from the medium, and as a result, the growth of the fungus is suppressed. In the transgenic lines expressing the siRNAs, the fungus can temporarily grow forming germ tubes and appressoria before penetration but could not invads into the rice cells (Figure 7). It is possibly that before the fungus invaded into the rice cells, the nutrients required for aerial hyphae and appressorium growth is provided by the spores. As a result, the hyphae and appressorium develop well. When the appressorium begins to invade the rice cells, the siRNAs generated from the hairpin constructs in the transgenic rice cells suppress *MoAP1* expression, leading to suppressed or delayed invasion. This conjecture needs to be further verified.

The asiRNAs targeting *MoAP1* may be used as an *in vitro* reagent to enhance rice blast resistance. This is consistent with the effects of asiRNAs in the other pathogens. For example, Koch et al. (2016) showed that the growth of *F. graminearum* was severely inhibited after spraying of target long dsRNAs on the surface of barley leaves. Wang et al. (2016) also showed that significant inhibition of gray mold disease could be obtained by applying asiRNAs or dsRNAs targeting *Botrytis DCL1* and *DCL2* genes on the surface of fruits, vegetables and flowers. Consequently, given the ease of design and applicability to diverse pathogens, the use of target-specific asiRNA as an anti-fungal agent offers unprecedented potential as a new plant protection strategy.

Taken together, we obtained a target gene and a target site that can be used in HIGS. Thus, this work may be relevant for the development of a strategy to engineer broad-spectrum blast-disease resistance in rice.

## Author Contributions

W-MW and HX have designed the research. X-YG performed the research with help from J-QZ. X-YG, YL, JF, and F-XX analyzed the experimental results. Y-FW and X-LC assisted with the inoculation test. J-QZ, JS, and YS assisted with the rice transgenic work. X-YG and W-MW wrote the manuscript.

## Conflict of Interest Statement

The authors declare that the research was conducted in the absence of any commercial or financial relationships that could be construed as a potential conflict of interest.

## Footnotes

1 http://www.broadinstitute.org
