# Supplementary material for: Host-Induced Gene Silencing of MoAP1 Confers Broad-Spectrum Resistance to Magnaporthe oryzae
Source: Front Plant Sci. 2019 Apr 9;10:433. doi: 10.3389/fpls.2019.00433 (PMC6465682; doi:10.3389/fpls.2019.00433)
Supplement: Supplementary file 1 [file Data_Sheet_1.docx]

**Supplementary Tables**

**Table S1 Disease assay on *MoAP1* -1362 transgenic lines with 11 *M. oryzae* strains***

| No. | Strains | Kasalath | *35S:MoAP1-1362 #24* | *35S: MoAP1-1362 #44* | Area |
| --- | --- | --- | --- | --- | --- |
|  |  | Resistance scale | | |  |
| M1 | Tepep | 5_S_ | 3 _S_ | 3 _S_ |  |
| M2 | GUY11 | 4_S_ | 2 _R_ | 3 _S_ |  |
| M3 | NC-14 | 5 _S_ | 2 _R_ | 3 _S_ | Sichuan province, China |
| M4 | NC-24 | 4 _S_ | 3_S_ | 3_S_ |  |
| M5 | WJ10 | 5_S_ | 3_S_ | 3s |  |
| M6 | Zhong1 | 5 _S_ | 3_S_ | 3_S_ |  |
| M7 | 97-75 | 5 _S_ | 2 _R_ | 3_S_ |  |
| M8 | NC-10 | 4 _S_ | 3_S_ | 3_S_ |  |
| M9 | B9 | 5 _S_ | 3_S_ | 2 _R_ |  |
| M10 | WQX | 4 _R_ | 2 _R_ | 3s |  |
| M11 | 089 | 4_S_ | 3_S_ | 3_S_ |  |

* The blast disease lesions were classified into five scales. Scale 0-2 is classified as resistance (R), and 3-5 classified as susceptibility (S) based on the sporulation rate on the lesions in infected rice leaves following a previous report (Bonman etal.,1986). In brief, 0 = no disease lesions observed; 1 = small pinpoint-like disease lesions between two small vascular bundles; 2 = lesions with diameter 0.5–1 mm and develop over the two small vascular bundles but do not reach the big vascular bundles; 3 = disease lesions with diameter about 1–3 mm and develop between the two big vascular bundles; 4 = disease lesions with diameter about 3–4 mm and develop over the two big vascular bundles; 5 = disease lesions with diameter over 4 mm and develop over the main vein.

**Table S2 Artificail small interference RNA synthesized in vitro**

| Gene | asiRNA | sense strand* | antisense strand* |
| --- | --- | --- | --- |
| *MoAP1* | 1245 | GCACAAAUCΜGAACGACAUTT | AΜGUCGUUCAGAUUΜGΜGCTT |
|  | 1362 | GCGGΜGACUUCUUUAACGATT | UCGUUAAAGAAGUCACCGCTT |
|  | 1115 | CCAGAGCCAUUCACACAAUTT | AUΜGΜGΜGAAΜGGCUCΜGGTT |
| *MoSSADH* | 735 | CAGGCGUΜGUCAACUUΜGUTT | ACAAAGUΜGACAACGCCΜGTT |
|  | 360 | GGΜGGUAΜGACCUCAΜGAUTT | AUCAΜGAGGUCAUACCACCTT |
|  | 1068 | GGGUCAAGGAAACGUUCAATT | UΜGAACGUUUCCUΜGACCCTT |
| *MoACT* | 91 | GACCGUCUUUCCΜGAAUCATT | ΜGAUUCAGGAAAGACGGUCTT |
|  | 622 | CCAGGACUUUAΜGCAAGGATT | UCCUΜGCAUAAAGUCCΜGGTT |
|  | 791 | GCUCUUUACUACCAAGAGUTT | ACUCUΜGGUAGUAAAGAGCTT |
| *MoSOM1* | 397 | GGUCAGCCAAUACAUUACATT | ΜGUAAΜGUAUΜGGCΜGACCTT |
|  | 682 | CCCUAGUUCAGCΜGACAAUTT | AUΜGUCAGCΜGAACUAGGGTT |
|  | 1879 | GUCCAAΜGCAAACCΜGAAUTT | AUUCAGGUUΜGCAUΜGGACTT |

*** M indicates either A, U, G, or C. Two T at the end of each strands stabilizes the RNA.**

**Table S3 Primers for qRT-PCR**

| Name of primers | Sequence |
| --- | --- |
| Moap1-F RT | ACTTGCAACAAGATTTGGGA |
| Moap1-R RT | TGAATTTTGG GGGCATCCCT |
| MoPot2-F for RT | ACGACCCGTCTTTACTTATTTGG |
| MoPot2-R for RT | AAGTAGCGTTGGTTTTGTTGGAT |
| ACTIN-F RT | TGCTATGTACGTCGCCATCCAG |
| ACTIN-R RT | AATGAGTAACCACGCTCCGTCA |

**Table S4 Primers for making RNAi constructs**

| Name of primers | Sequence |
| --- | --- |
| *MoAP1*-1245-RNAi-F | TCGACTCGCTCTTCGACAAC |
| *MoAP1*-1245-RNAi-R | TGTTTTGCGCCTTGCGCTTC |
| *MoAP1-*1362-RNAi-F | ATCTGAACGACATCGACTGG |
| *MoAP1*-1362-RNAi-R | TTGACGTCGACCGCATCATC |
| *MoAP1*-1115-RNAi-F | ATCGAGGAGCAGCATGGACT |
| *MoAP1*-1115-RNAi-R | TGCTCGAGAAGTGGTCTAAA |
| Full-length *MoAP1*-F | ACAGGTACC ATGTTGACCCCGC AGCAACAGAGTCT |
| Full-length *MoAP1*-R | ACAGGTACCGCTCGACGTCGCC  GCACCTGAATTTT |


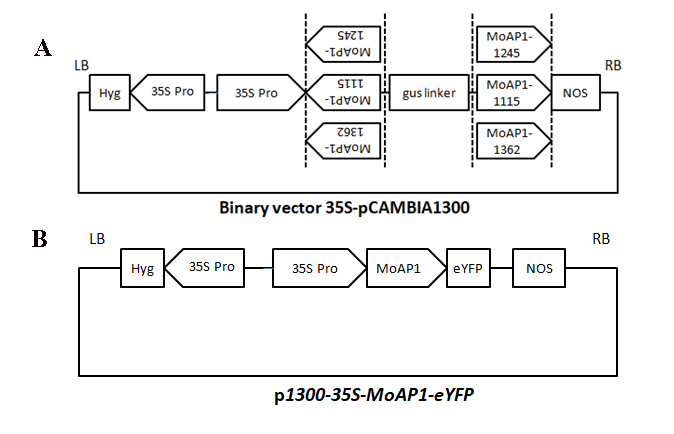


**Figure S1 Vectors construction. Diagrams show the construction of** the pANDA-*MoAP1*-RNAi plasmids (i.e., pANDA-*MoAP1-1115*(1115i), pANDA-*MoAP1-1245*(1245i), and pANDA-*MoAP1-1362*(1362i) (A) and *MoAP1-eYFP* chimeric gene (B).

**
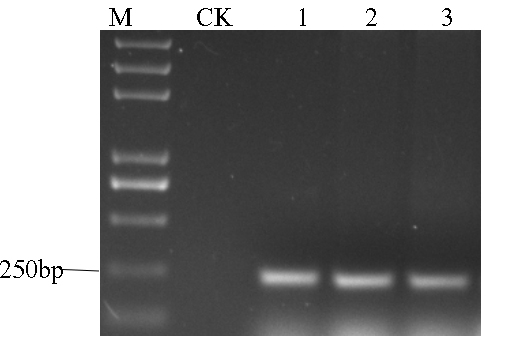
**

**Figure S2 PCR amplification shows the present of asiRNA1362 in T0 transgenic plants.** M：DNA marker 2000；CK：Negative control；1-3 : Transgenic rice carrying pANDA-MoAP1-1362-RNAi construct.
